# Recent Advances in Synthesis and Application of Metal Oxide Nanostructures in Chemical Sensors and Biosensors

**DOI:** 10.3390/nano12244413

**Published:** 2022-12-10

**Authors:** Vincentas Maciulis, Almira Ramanaviciene, Ieva Plikusiene

**Affiliations:** 1 State Research Institute Centre for Physical Sciences and Technology, Sauletekio Ave. 3, LT-10257 Vilnius, Lithuania; 2 Nanotechnas–Center of Nanotechnology and Materials Science, Faculty of Chemistry and Geosciences, Vilnius University, Naugarduko Str. 24, LT-03225 Vilnius, Lithuania

**Keywords:** nanostructures, biosensors, chemosensors, metal oxides

## Abstract

Nanostructured materials formed from metal oxides offer a number of advantages, such as large surface area, improved mechanical and other physical properties, as well as adjustable electronic properties that are important in the development and application of chemical sensors and biosensor design. Nanostructures are classified using the dimensions of the nanostructure itself and their components. In this review, various types of nanostructures classified as 0D, 1D, 2D, and 3D that were successfully applied in chemical sensors and biosensors, and formed from metal oxides using different synthesis methods, are discussed. In particular, significant attention is paid to detailed analysis and future prospects of the synthesis methods of metal oxide nanostructures and their integration in chemical sensors and biosensor design.

## 1. Introduction

Nanostructured materials offer a number of advantages, such as large surface area, improved mechanical and other physical properties, as well as adjustable electronic properties that are important in the development of various sensors [1,2,3,4,5,6,7,8]. Properties of nanostructures differ greatly when compared to bulk materials. New material properties appear at nanometer dimensions, and a greater amount of atoms are found on the surface of the nanoparticle compared to higher volume particles. Therefore, the size is inversely proportional to the surface area and its surface-to-volume ratio [9]. Nanostructures are classified using the dimensions of the nanostructure itself and their components. Nanostructure classes were built using the comprising elementary units, namely, 0D nanoclusters and nanoparticles, 1D nanotubes and nanowires, and 2D nanoplates and nanolayers [10]. Nanostructures where the particles are smaller than 100 nm in all relevant directions can be referred to as ‘0D nanostructures’. These nanostructures include nanoclusters, nanospheres and fluorescent semiconductor nanocrystals—quantum dots [11,12,13]. For example, ZnO nanospheres can be prepared from zinc acetate in an alcoholic solution under basic conditions; however, the shape of the particles is very sensitive to the concentration of precursor materials [14]. Therefore, to allow some nanoparticle size control and protection from aggregation, 0D nanostructures must be synthesized from micelles or within hard templates. One avenue for the formation of 0D nanostructures is the use of soft templates, surfactants, or polymers of well-defined structure, dendrimers. Alternatively, surface masking or patterning can also be used, applying different lithographic approaches [15]. One-dimensional nanostructures are structures that have lateral dimensions greater than 100 nm, which can be formed using different metals and metal oxides and obtain desirable morphology, such as nanowires, nanorods, nanotubes, nanobelts, and nanoribbons [16,17,18,19]. Because of their unique properties and potential in application for many fields such as batteries, solar cells, gas sensors and biosensing, these materials are being studied in great detail [20,21,22]. Various synthesis approaches are available for the formation of 1D nanostructures, electrospinning, electrochemical deposition/anodization, chemical/physical vapor deposition, and chemical bath deposition [23]. Two-dimensional nanostructures are two-dimensional structures, such as nanofilms, nanocoatings, nanolayers, and nanowalls. All of these structures have a large surface-to-volume ratio and confined thickness, which is highly desirable in applications such as catalysis, photovoltaics, and sensing [24,25,26,27]. Example methods to synthesize 2D materials include: liquid exfoliation, chemical vapor deposition (CVD), and atomic layer deposition (ALD) [28]. The nanostructures named ‘3D nanostructures’ are made of lower dimensional structures (0D, 1D, 2D); thus, the 3D nanostructures have the most varied morphology out of all classes of nanostructures, and the morphology includes structures containing rods, cubes, spheres, etc. [29,30,31]. Although 3D nanostructures are not bound to the nanoscale, just as bulk materials, they still have the effect of being made of nanostructures (quantum confinement effect, high surface-to-volume ratio) [32]. Chemical synthesis is the most straightforward way of forming 3D nanostructures applying methods of CVD, vapor liquid solid (VLS), electrodeposition, and electrochemical etching [33,34,35,36,37]. Nanostructures of a wide variety of materials are applied for sensing technologies due to increased surface area and tuneable properties for sensor signal enhancement.

Hence, the present review deals with various types of metal oxide nanostructured synthesis methods. In particular, the review presents detailed analysis and future prospects on the synthesis methods of 0D, 1D, 2D, and 3D nanostructures and their integration in sensors and biosensor design.

## 2. Nanostructure Synthesis Methods

In this review, we present selected synthesis methods most commonly used to form metal oxide nanostructures applied for chemical sensing and biosensing. In the last decade, a large number of publications were dedicated to the presented synthesis methods and for the development of desirable properties from 0D to 3D metal oxide nanostructures. Metal oxide nanostructures can be made as a result of a simple precipitation chemical reaction. These syntheses can be referred to as ‘wet chemical’ methods. However, it can be difficult to achieve precise dimensions and novel morphology of nanostructures using wet chemical methods alone. Therefore, the application of templates, external energy (thermal or electrical), and pressure can lead to more control and more variation in the morphology of the formed metal oxide nanostructures. The presented methods are suitable for the fabrication of metal oxide nanostructures of different sizes and morphologies, which can be used for various target analytes in the development of sensors.

### 2.1. Reverse Microemulsion Method for the Synthesis of Nanostructures

**Figure 1:**
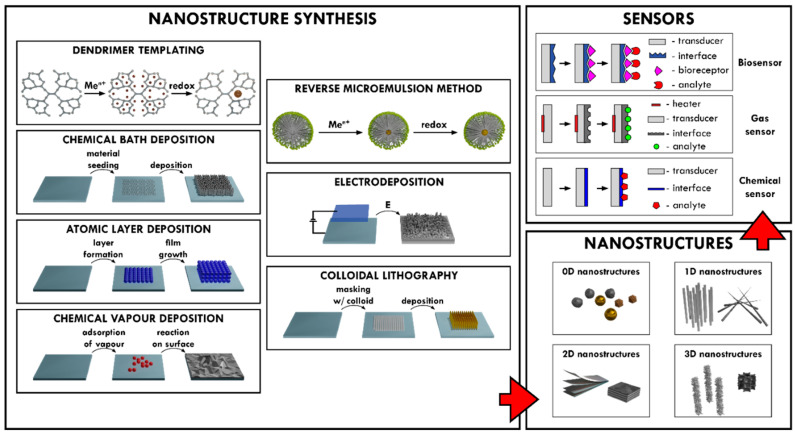
Synthesis methods of various nanostructures and their application in different sensors design.

Synthesis in reverse microemulsions is an avenue for the formation of 0D nanostructures. Microemulsion is an isotropic and thermodynamically stable system of two non-miscible liquids and a surfactant [38]. Depending on the polar/nonpolar fluid ratio, surfactants can be thermodynamically driven to self-assembly generating micelles (in polar surroundings) or reverse micelles (in non-polar surroundings). Usually, 0D nanostructure synthesis is undertaken in reverse micelles, sometimes described as ‘nanoreactors’ [39,40]. This allows the polar and ionic compounds to be separated into isolation from nonpolar surroundings, inside the hydrophilic centers of the reverse micelles. Micelles can, and frequently do, interact with each other, colliding, breaking apart, and exchanging trapped compounds; this is what drives nucleation and growth [41]. The methods for nanostructure synthesis are presented in Figure 1.

In a work by Du et al., 2012, NiO nanoparticles were synthesized in reverse micelles by reducing NiCl_2_ with aqueous NH_3_ and calcinated in a furnace. Microemulsion consisted of oil (cyclohexane)/surfactant (Triton X-100)/cosurfactant (n-hexanol)/water (NiCl_2_ + NH_3_). By varying the proportions of microemulsion constituent parts, mixing method and calcination temperature, the particle size of NiO was controlled from 11.5 to 31.5 nm. The smallest NiO nanoparticles were synthesized in 44% cyclohexane: 33% Triton X-100: 16% n-hexanol: 7% water solution (inorganic precursors), mixing the microemulsions in a dropwise manner, calcinating in 450 °C for 2 h. Controlling particle size is crucial for sensing application, as lowering the diameter of the NiO particles increased the sensitivity to H_2_S gas when measured at 150 °C [42]. However, not all syntheses methods require mixing two separate microemulsions to form nanoparticles. Liang et al., 2017, synthesized ultrafine α-Fe_2_O_3_ nanoparticles using the microemulsion method. The solution consisted of Fe(NO_3_)_3_, NaCl, deionised water, CTAB, benzene, and was stirred at 70 °C for 6 h. The temperature was then increased to 90 °C for azeotrope distillation to remove the water. The dried sample was calcinated for 5 h at 500 °C. The resultant particles have a narrow distribution, spanning from 2.3 to 3.9 nm [43]. Ali et al., 2019, produced well defined V-doped ZnO nanoparticles by microemulsion, with a tunable band gap based on incorporated vanadium amount [44].

### 2.2. Dendrimer Templating for Nanostructure Formation

Dendrimers are monodisperse macromolecular structures with extending branches from the center, highly symmetrical and exhibit 3D or 0D morphology, which is essential for not only the formation but the stabilization of 0D nanostructures [45,46]. Generally, for metal nanocluster synthesis, metal ions are incorporated inside the dendrimer structure by coordinative interaction with functional groups of dendrimer interior. Then, trapped ions are chemically reduced to form nanoclusters with a defined number of atoms. The number of atoms in a cluster can be controlled by changing the encapsulating dendrimer [47].

**Figure 2:**
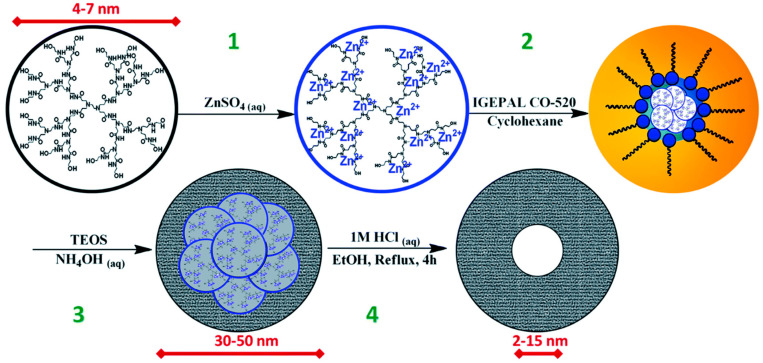
Scheme for total synthesis of dendrimer encapsulated mesoporous silica NPs. Step (1) inoculation of dendrimer with metal ions, (2) formation of reverse microemulsion with disperse phase (blue) and continuous phase (orange), (3) base catalyzed silica formation, and (4) acid catalyzed etching of metal encapsulated dendrimers. Adapted from [45].

Dendrimers are used not only for 0D metal oxide nanostructure synthesis but also as stabilizers. In the study by Nakanishi and Imae, 2005, TiO_2_ nanoparticles were formed by hydrolysis of TiCl_4_ in water with and without dendrimers. Liquid TiCl_4_ (−20 °C) was added to a solution at pH 10 in the presence and absence of G4.5-COONa dendrimer under stirring and maintained 0 °C. TiO_2_ nanoparticles were evaluated and their diameters compared. The TiO_2_ nanoparticles synthesized without the use of dendrimers were larger and dependent on the pH of the solution, while the average size of the particles formed inside the dendrimers was smaller (7.5 nm for non-dendrimers and 4.4 to 6.7 nm for dendrimer-protected) and the particle size depended on the dendrimer terminal groups. Additionally, suspensions of dendrimer-protected nanoparticles were stable over several months, as the dendrimer coating prevents TiO_2_ NPs from aggregating [48]. A comparative study was conducted by Vijayalakshmi et al., 2020, on stabilizing agents of polyvinylpyrrolidone (PVP), glycodendrimer, and chalcone dendrimer for Ag@SnO_2_ nanoparticle synthesis. Ag core-SnO_2_ shell nanoparticles were synthesized by the redox-transmetalation method, using stabilizing agents that result in nanoparticles of 30–40 nm diameter with similar structural parameters and indirect band gap energy of 3.84–4.0 eV. Photoluminescence studies identified that Ag@SnO_2_ nanoparticles emitted photons in UV. The antibacterial and antifungal properties were tested with mixed results [49]. Dendrimer templating can also be used as a part in multi-step synthesis. The following development of microporous SiO_2_ nanoparticles was published by Rosenberg et al., 2019: microporous SiO_2_ nanoparticles were synthesized by silanization in the reverse microemulsion of Zn^2+^- and Cu^2+^-loaded dendrimers. A scheme for total synthesis is depicted in Figure 2. The composition of the reverse microemulsion system, in addition to varying the dendrimer generation, allowed for the control of microporous SiO_2_ nanoparticle generation with a particle size of 20–50 nm and a micropore size of 2–15 nm. Dendrimers loaded with Cu^2+^ produced larger micropores than Zn^2+^, while empty dendrimers did not provide micropore formation.

### 2.3. Chemical Bath Deposition (CBD) for Nanostructure Synthesis

**Figure 3:**
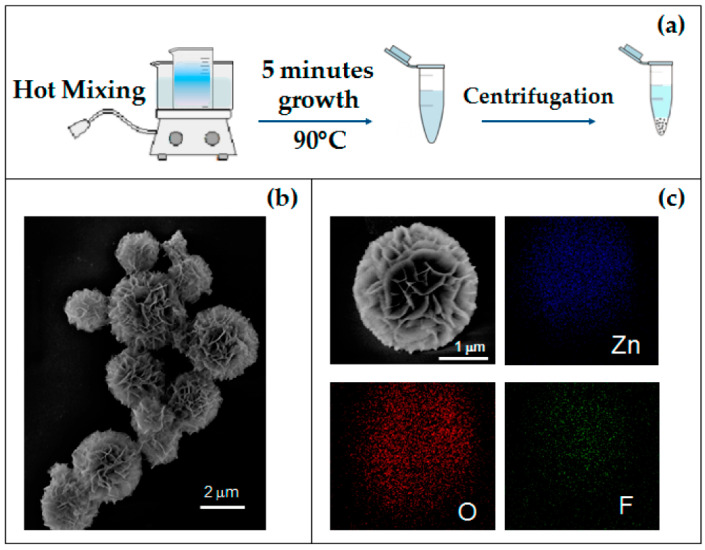
(**a**) Schematic synthesis diagram of the 3D flower-like hierarchical ZnO microstructures; (**b**) SEM image of as-grown MFs; (**c**) SEM image of a single MF and corresponding EDX elemental maps. Adapted from [56].

CBD can be considered to be the simplest method for depositing films of semiconductors or metal oxides. The procedure is generally performed from aqueous solutions where the material is generated and deposited on the substrate in the same bath. The method requires minimal substrate preparation and can be used to deposit films on any substrate as long as it is chemically stable while in deposition solution. Glass is a common choice when transparency is necessary [50]. Plastics can also be used as a substrates; however, their adhesive properties to deposited material can vary, meaning surface treatment before deposition may be required [51,52]. Monolayers (for example, silanes) with specific functional groups can be used to coat the substrate and direct the deposition toward (with hydrophilic groups) or away from (with hydrophobic groups) specific areas in an attempt to create patterned surface depositions [53,54,55]. ZnO nanostructures, named microflowers (MFs), have been formed by a chemical bath deposition method by Strano et al. The formed nanostructures are presented in Figure 3 [56].

CBD can be utilized to form films on substrates of any shape. In the work by Chua et al., 2021, MoO_3_ nanorods were deposited on tapered optical fiber for room temperature ammonia sensors. The deposition was carried out in an aqueous solution mixture of 0.1 M Na_2_MoO_4_ and 0.65 M HNO_3_ solution at 85 °C, in a two-step synthesis. First, the optical fibers were immersed in synthesis solution for 20 min to form MoO_3_ nucleates, rinsed with deionized water and placed back in synthesis solution for 10 min to grow MoO_3_ nanorods. Optic fiber with MoO_3_ deposition was then annealed. Few annealing temperatures were chosen, with 150 °C having the best results for optical ammonia sensor [57]. Nanostructures of ZnO are widely deposited using the chemical bath deposition method. In this work by Vessalli et al., 2017, ZnO nanorods (ZnO NRs) were grown with graphene oxide (GO) to form a composite material. The ZnO NRs were deposited in Zn(NO_3_)_2_ HMTA solution: (1) on ZnO seed layer, (2) on GO layer, and (3) with added GO in solution. This resulted in three different sensors to volatile organic compounds (VOCs) of varied selectivity. This change in selectivity is explained by the sensor’s change in resistance when GO is deposited under ZnO NRs. When the sensor was modified with GO deposition under and on ZnO NRs, better sensor selectivity was achieved [58]. Husham et al., 2017, introduced a synthesis of ZnO NRs from Zn(NO_3_)_2_ and HMTA solution using microwave-assisted CBD to form a metal-semiconductor-metal-based UV sensor. The ZnO seeding layer was deposited by RF sputtering on clean Si substrate and annealed at 400 °C for 1 h. ZnO NRs were grown in Zn(NO_3_)_2_, HMTA solution at 90 °C for 2 h, heated by microwave heating. Aluminum and palladium contacts were deposited to utilize ZnO NRs for UV sensing. Grown ZnO NRs show vertical alignment and low defect density. A device based on ZnO NRs show remarkable potential for low-/self-powered sensor [59]. Gas sensing properties of CBD-formed ZnO nanostructures are also studied widely [60,61,62].

### 2.4. Electrodeposition for the Formation of Nanostructures

**Figure 4:**
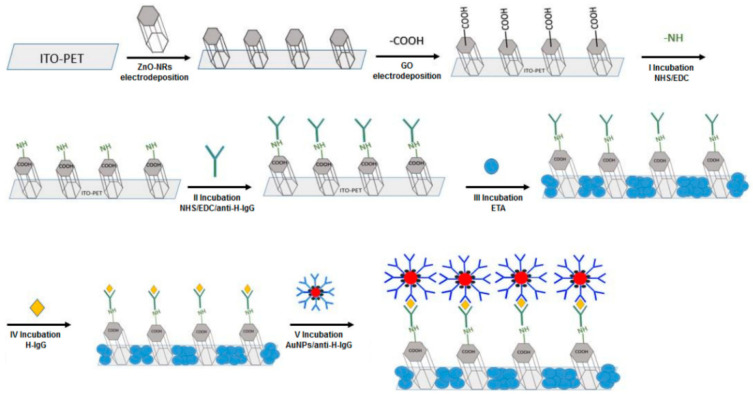
Scheme of the fabrication of immunosensor with a sandwich configuration based on ZnO nanorods. Adapted from [67].

As a deposition method, electrodeposition has many advantages compared to other methods, such as catalyst-free process, a short deposition time, a low deposition temperature and the ability to make homogeneous large-area depositions [63,64]. The flexibility of the electrodeposition method for the formation of ZnO nanostructures was fully shown in a study by Chen et al., 2013. The ZnO nanostructure deposition from the Zn(NO_3_)_2_ electrolyte with KCl was analyzed by looking at the effects of Zn(NO_3_)_2_, KCl concentrations, deposition temperature and deposition voltage. An increase in KCl supporting electrolyte concentration from 0 to 1 M created ZnO depositions of varied structures from microspheres (0.03 M KCl) to nanosheets (0.1–1 M KCl). Running an electrodeposition with high KCl concentrations also forms zinc hydroxyl compounds alongside ZnO, with the decomposition of the former in high temperatures potentially leading to the formation of porous ZnO nanosheets. An increase in the concentration from 0.005 to 0.5 M creates ZnO depositions from nanospikes (0.005–0.02 M) to nanosheets (0.1–0.5 M). Deposition temperature also has an effect on electrodeposited nanostructures, with deposited structures changing from nanowires to nanosheets with increasing temperature. Increasing the deposition potential seems to increase the density of nanostructures; it also promotes the formation of zinc hydroxyl compounds along with ZnO [65]. Mollarasouli et al., 2020, used the two-step electrodeposition method to form NiWO_4_ nanostructures for non-enzymatic glucose sensor. The first step was the deposition of Ni-W metal alloy under –1.4 V for 60 s using 0.67 mM NiSO_4_, 0.3 mM Na_3_WO_4_, 0.26 mM citric acid, and 31 mM Na_2_SO_3_. The pH was adjusted to 8.0, for the citrate to form complex with Ni and W. The second step involved formation of NiWO_4_ from deposited Ni-W alloy by cyclic voltammetry in 1 M NaOH for 15 cycles at 50 mV s^−1^. CV treatment under basic conditions with low scan rate is required for the diffusion of OH^−^ anions to the Ni-W alloy [66]. Patella et al., 2022, developed an immunosensor based on ZnO NRs fabricated by electrodeposition. Indium tin oxide on polyethylene terephthalate (ITO-PET) was used as a flexible substrate and ZnO NRs were deposited with optimized conditions of −0.95 V vs. Ag/AgCl, for 60 min in 10 mM ZnCl_2_ and 10 mM NaNO_3_ at pH 4.5, resulting in hexagonal ZnO NRs with 800 nm mean length. The schematic representation of ZnO nanorods formed using the electrodeposition method and application in sandwich format immunosensor designed for human immunoglobulin G detection is presented in Figure 4 [67].

### 2.5. Chemical Vapor Deposition (CVD) for Synthesis of Nanostructures

**Figure 5:**
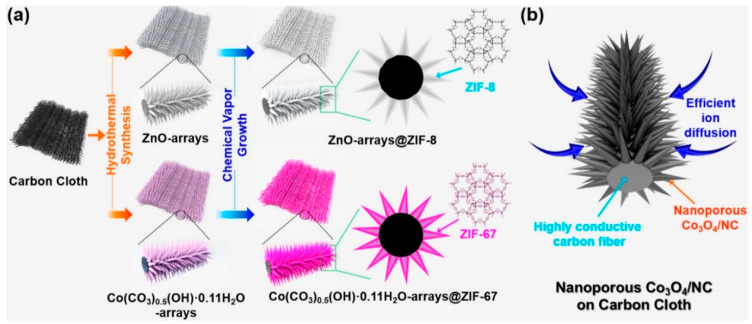
Scheme showing the synthetic process to generate (**a**) ZnO@ZIF-8-x-y and Co(CO_3_)_0.5_(OH)·0.11H_2_O@ZIF-67-x-y using 2-Melm vapor, where x and y are the synthesis temperature and synthesis time, respectively. (**b**) Advantages of Co_3_O_4_/NC hybrid materials. Adapted from [34], 2018, American Chemical Society.

It is a well-known and widely applied technique for solid phase material formation [68,69,70,71]. Unlike physical vapor deposition, which requires sputtering or evaporation, CVD is a method in which the precursor material is thermally decomposed or chemically reacts on the substrate and is allowed to grow, producing a thin film. The growth of thin films is regulated by temperature, concentration of reactants, and pressure [72]. There are modified CVD procedures, for example: plasma-enhanced chemical vapor deposition (PECVD), where plasma is created of the reacting gases and deposition occurs, low-pressure chemical vapor deposition (LPCVD) and atmospheric pressure chemical vapor deposition (APCVD) [73,74,75]. Deposition using CVD on substrates with 3D morphology has been tested and offered promising applications. A process for metal-organic-framework growth on carbon cloth was tested. ZnO and cobalt carbonate hydroxide (Co(CO_3_)0.5(OH)∙0.11H_2_O) were deposited on carbon cloth. Using CVD, the ZnO layer was converted into ZnO@ZIF-8 using 2-methylimidazole (2-Melm) vapor at 100 °C, while (Co(CO_3_)_0.5_(OH)∙0.11H_2_O) was converted into Co(CO_3_)_0.5_(OH)·0.11H_2_O@ZIF-67. Subsequent heat treatment for the Co(CO3)_0.5_(OH) < 0.11H_2_O sample resulted in the formation of CoO_3_ and nanoporous carbon (CoO_3_/NC), creating an electrode exhibiting excellent electrochemical performance (Figure 5).

In a work by Barreca et al., 2010, 1D ZnO nanostructures of high aspect ratio were grown on alumina substrates at 300 °C by PECVD for the application of CO, H_2_ and CH_4_ gas sensors. CVD precursors were obtained by reaction of diethyl zinc with neutral ketoiminate in hexane. For CO and CH_4_, best sensing temperature was 200 and 300 °C, respectively. For H_2_ gases, the sensing ability increased with increasing temperature, with the best response to H_2_ gases at a maximum measured temperature of 400 °C [76]. Zhang et al., 2012, produced a gas sensor for NO_2_, CH_4_ and CO based on 1D SnO_2_ nanobelts formed from high-purity Sn powder modified with gold (Au). SnO_2_ nanobelts were formed using the water-assisted CVD method at 850 and 1000 °C. Au modification plays a crucial role in the formation of the SnO_2_ nanobelts as a structure directing agent [77]. A modification of quartz crystal microbalance (QCM) sensor was published by Wu et al., 2020, Mg-ZnO nanostructures were directly grown by MOCVD at 500 °C using diethyl zinc, bis-methyl-cyclopentadienyl magnesium, and oxygen, resulting in uniform NRs of 405 nm in average height. Formation of 1D nanostructures greatly increased the surface area of the sensor, and Mg inclusion to the nanostructure stabilizes the ZnO denying the release of toxic Zn^2+^ ions, thus minimizing the toxicity to microorganisms [78]. A gas sensor for ethanol and acetone was formed on fluorine-doped and undoped Co_3_O_4_ nanodeposits grown on alumina substrates by Barreca et al., 2011. Two precursors enabled the synthesis using PECVD of fluorine-doped and undoped Co_3_O_4_. This resulted in a favorable influence of fluorine on the Co_3_O_4_ system. The introduction of fluorine into deposition created a more responsive gas sensor and lowered its working temperature, both features are highly regarded in technological application [79]. 

### 2.6. Atomic Layer Deposition (ALD) Method for the Formation of Nanostructures

ALD is a subclass of CVD in which the process is self-limiting and requires the separation of precursor materials and the introduction to the substrate in sequence [80]. Deposition happens in cycles with a given amount of material deposited per cycle, from less than 0.2 to 12 nm per cycle [81]. ALD can be applied to form thin 2D metal oxide nanostructures that usually are amorphous due to low deposition temperatures, and require post deposition treatment or deposition at a higher temperatures [82]. ALD can be applied for deposition on more complex substrates (2D, 3D nanostructures) and on variety of materials: metals, glasses, oxides, graphene, and polymers [83,84,85,86,87,88]. Water-soluble polymers can be coated with ALD and used as sacrificial layers to form free-floating 2D structures [89]. ALD is considered to be the main method of choice for conformal deposition of thin film/monolayer metal oxide, sulphide, and nitride formation on complex nanostructures, despite the wasteful usage of energy and reagents [80,90].

**Figure 6:**
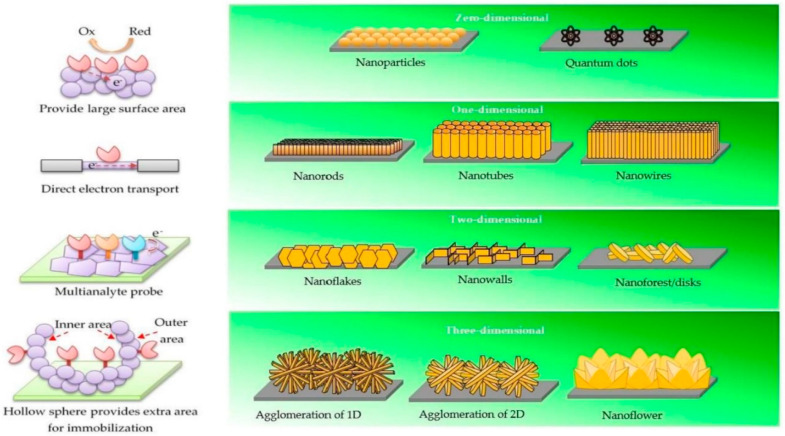
Four different dimensions of ZnO nanostructures with their advantages. Zero-dimensional nanostructures provide a large surface area. One-dimensional nanostructures possess stable and direct electron transport. Two-dimensional nanostructures give specific planes for immobilization process for the simultaneous detection of different analytes. Three-dimensional nanostructures have extra surface area (outer and inner area) to provide more sites for immobilization. Adopted from [27].

As mentioned above, ALD-based nanostructures and nanolayer depositions can be used on complex nanostructures for the uniform deposition of functional material. Recently, ZnO nanostructure application in biosensor design became a hot topic. ZnO has high isoelectric point and this feature makes it a good candidate for biosensor applications. Moreover, it can be mass produced due to its cost-effectiveness, it is nontoxic, it is chemically stable and different nanostructures can be formed. The different dimensions of the ZnO nanostructures with their advantages are presented in Figure 6 [27].

Chaaya et al., 2014, proposed the deposition of ZnO thin films by ALD on electrospun poly(acrylonitrile) (PAN) fibers, to enhance the UV detection capabilities. Diethyl zinc (DEZ) and H_2_O were used as precursors for the deposition of ZnO ALD. The effects on the sensitivity of the UV sensor of the deposition cycle number, temperature, and electrospinning increased the photoresponsive current of UV by a factor of 250 compared to a flat electrode. This increase in sensitivity comes from an increase in surface area, with smaller grain sizes having a more positive UV photoresponse [16]. The ALD technique has advantages in comparison to other chemical deposition methods, namely, the deposited material is conformal, pinhole-free, has good adhesion to the substrate and is suitable for periodic nanostructures, such as nanolaminates, and periodic coating of multiple materials of thickness in the nanoscale formations [16,91,92,93,94]. In the article by Balevicius et al., 2018, Al_2_O_3_/ZnO nanolaminates were formed by ALD and tested for optical biosensor application using total internal reflection ellipsometry (TIRE). Nanolaminates were formed on glass substrates from DEZ and H_2_O for ZnO and trimethylaluminium (TMA) and H_2_O for Al_2_O_3_, and deposition was performed at fixed 100 °C. The samples had a total thickness of 200 nm, consisting of (i) four alternating Al_2_O_3_ and ZnO layers of 50 nm thickness and (ii) two Al_2_O_3_ and ZnO layers of 100 nm thickness. When comparing the two nanolaminate structures, a higher sensitivity was achieved using 50 nm layers of Al_2_O_3_ and ZnO. This increased sensitivity was explained by multiple reflections from the layer boundaries [1].

Lou et al., 2021, proposed the use of ALD for the formation and application of formaldehyde detection. Hydrothermally synthesized powdered SnO_2_ nanospheres were coated with ZnO using ALD at 180 °C using DEZ and H_2_O, and they were annealed at 400 °C for 2 h. SnO_2_/ZnO heterojunction formation using ALD created a superior nanosphere gas sensor compared to SnO_2_ nanospheres, the optimal working temperature was lowered by 40 °C and reached 200 °C, and sensor response was increased by a factor of 7 [95].

## 3. Application of Metal Oxide Nanostructures for Chemical Sensing and Biosensing

In 1962, Clark and Lyons presented the idea of the biosensor and suggested that enzymes may be immobilized on electrical detectors to create enzyme electrodes. Using glucose oxidase (GOx), the first enzyme electrode was developed to track blood glucose levels [96]. A biosensor is an analytical system consisting of a biological sensing component connected to a transducer that transforms biochemical reactions into an electrical or optical signal that is proportional to the concentration of the targeted analyte.

Biosensor technology has received interest in the last decade and has been applied in many areas: food, environment, and healthcare [97,98]. Biosensors have a very broad definition; they can be classified by bioreceptor type (enzyme, antibody, DNA, cells, biomimetic) and by signal transduction (optical, calorimetric, piezoelectric, electrochemical) [99]. There is plenty of application for nanostructures to be utilized in signal transducing as signal enhancers. The nanostructure formation methods and application in biosensor design are summarized in Table 1.

Faria and Mazon, 2019, fabricated electrochemical immunosensors based on ZnO nanowires (NWs) to diagnose early-stage Zika virus infection from patient urine. ZnO NWs were deposited using the CBD method on a graphene-modified Au printed circuit board. Such modification has potential in lowering the limit of detection (LOD) by increasing the surface area of the electrode, as well as providing a surface with high chemical stability, thus having a suitable surface for antibody immobilization. Monoclonal Zika virus antibodies (ZIKV-NS1) were immobilized on the ZnO NW using cystamine (Cys) and glutaraldehyde (GA), and a diluted solution of antibodies was dropped on the surface and left to incubate for 12 h. Calibration of the sensor was performed, achieving good linearity in the 0.1–100 ng/mL range of ZIKV-NS1 and a low LOD of 1 pg/mL, showing potential in application as a rapid point-of-care test [100].

**Figure 7:**
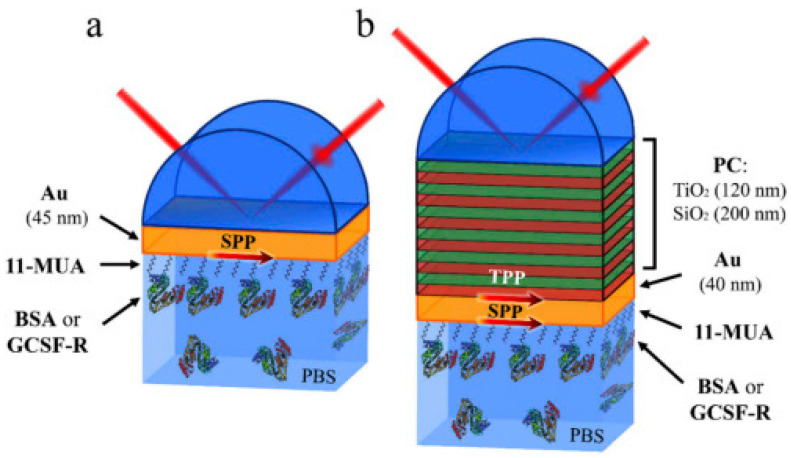
Total internal reflection geometry schematic of the Au (a) and PC/Au (b) samples with a self-assembled monolayer of 11-mercaptoundecanoic acid (11-MUA) and the GCSF-R or BSA protein in phosphate-buffered saline solution. Adopted from [105].

In the work by Buzavaite-Verteliene et al., 2020, the TIRE method was used for the study of the excitation and sensitivity properties of the hybrid Tamm plasmon polariton-surface plasmon polariton (TPP-SPP) and single surface plasmon resonance (SPR) modes of immobilization of bovine serum albumin (BSA) and the granulocyte colony stimulating factor receptor (GCSF-R) on the surface. The plasmonic photonic nanostructure was formed from high (TiO_2_) and low (SiO_2_) refractive index bilayers covered by a thin (40 nm) Au layer. The SPP component (δΔ_h-SPP_/δλ = 53.9°/nm) of the hybrid TPP-SPP mode showed 6.4 times higher sensitivity than the single SPR (δΔ_SPR_/δλ = 8.4°/nm) during the formation of the bovine serum albumin (BSA) layer on the Au film. The sensitivity using the hybrid plasmonic mode was found to be controlled by using the strong coupling effect between the components of TPP and SPP. Two optical sensor geometries are presented in Figure 7 [105].

**Figure 8:**
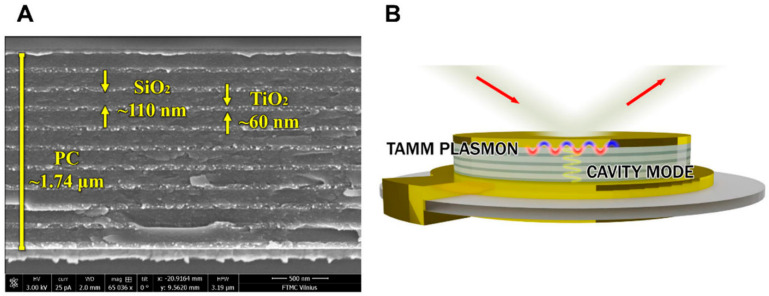
SEM micrograph of the plasmonic photonic structure modified QCM-D sensor chip (**A**) and Tamm plasmons and cavity mode excitation using nanometer structures of formed photonic crystal (**B**). Adopted from [106].

In the work by Plikusiene et al., 2021, the formation of 1D plasmonic photonic structures from TiO_2_ and SiO_2_ nanolayers on the commercially available quartz crystal microbalance with dissipation (QCM-D) sensors chip was reported. This thin Au film is able to generate Tamm plasmon polaritons and cavity modes that can enhance the optical signal of spectroscopic ellipsometry (SE) without using a coupler, as is usually necessary for the excitation of surface waves applied for biosensing [109,110,111,112]. The scanning electron microscope (SEM) micrograph of the nanostructures used for Tamm plasmons and cavity mode (CM) excitation, formed on the QCM-D sensor disc, is presented in Figure 8.

The Tamm plasmon optical state and CM for the modified QCM-D sensor disc showed sensitivity of ellipsometric parameters to refractive index unit (RIU) as follows: Ψ_TPP_ = 126.78 RIU^−1^ and Δ_TPP_ = 325 RIU^−1^, and Ψ_CM_ = 264 RIU^−1^ and Δ_CM_ = 645 RIU^−1^, respectively. This study shows that Tamm plasmon and CM have 23 and 49 times better performance of ellipsometric parameters, respectively, for refractive index sensing than standard SE signal on a QCM-D sensor chip [106].

A chemical sensor is a system that produces a useful signal as a result of a chemical interaction, both qualitative and quantitative. This system contains a transducer domain and a chemical interface layer. At a chemical interface, an analyte chemically reacts with a surface and creates a change in chemical or physical properties; this change is then measured by the transducer and generates an electrical signal proportional to the analyte concentration. Chemical sensors are classified by their transduction method (optical (refractometric, ellipsometric), electrical (conductometry, amperometry), and mechanical (QCM-D)) as well as their structure/composition of the chemical interface layer (metal, metal oxide, and metal semiconductor) [113]. The summary on nanostructures used for chemical sensing is presented in Table 2.

Rahman et al., 2020, published work on modifying a glassy carbon electrode (GCE) with nickel-doped ZnO (Ni-ZnO) nanostructures in Nafion solution to form an electrochemical hydrazine sensor. A three-electrode system was used for electrochemical analysis, and Ag/AgCl, Ni-ZnO/Nafion/GCE, and Pt were used as reference, working, and counter electrodes, respectively. The Ni-ZnO/Nafion/GCE sensor for hydrazine detection was employed in the phosphate buffer system in room-temperature conditions. The sensor exhibited excellent sensitivity with a linear response from 0.2 nM to 0.02 M with an LOD of 1.7 pM. This sensitivity can be explained by the hydrazine detection mechanism. When the Ni-ZnO surface is exposed to hydrazine, surface-mediated hydrazine oxidation takes place, producing N_2_H_4_ decomposition and delivery of electrons onto Ni-ZnO; at the same time, OH^−^ removal lowers the conduction band of Ni-ZnO, yielding a shift of the current during electrochemical investigation. This shift effect, along with an injection from hydrazine oxidation, produces an increased number of electrons, enhancing the conductance of the electrode. A real sample analysis of industrial effluent water and wastewater showed ~100% recovery of hydrazine from sample solutions, thus concluding its suitability to determine hydrazine in real aqueous samples [122].

In a study by Shetti et al., 2019, an electrochemical sensor was manufactured using Ag-doped TiO_2_ nanoparticles (Ag-TiO_2_ NPs) mixed with multi-walled carbon nanotubes (MWCNTs) for the detection of cetirizine, an anti-inflammatory drug. The electrochemical sensor is based on the modified carbon paste electrode, by blending MWCNTs and Ag-TiO_2_ NPs together with graphite powder and homogenizing with paraffin oil. This results in a sensor that exhibits a higher peak current with a less positive potential for cetirizine oxidation than for an unmodified electrode; however, the oxidation of cetirizine was found to be irreversible. Both Ag-TiO_2_ and MWCNT have a positive effect on enrichment in cetirizine oxidation, playing significant roles in the fast electron transfer process. This enables the sensor to detect cetirizine in very low amounts with LDR from 0.3 to 3 μM and an LOD of 8.76 nM [118].

## 4. Conclusions

Recently, progress and advances in the synthesis methods and application area of various metal oxide nanostructures illustrate the great relevance and necessity of such structures in the design of different types of sensors. The present review summarizes progress and advances in the synthesis methods of various 0D, 1D, 2D, and 3D metal oxide nanostructures and their application in chemical sensors and biosensors for different target analytes’ detection.

The 0D nanostructures, formed by microemulsion or co-precipitation/reduction methods, can be successfully applied in highly sensitive chemical sensors and biosensor design. Efficient synthesis of 0D nanostructures lies in the control of the nanoparticle formation process. However, these methods usually require expensive, complex or wasteful protocols; thus, more simple methods with less size control tend to be preferred. Simple, time-saving and convenient methods of synthesis, such as chemical bath deposition, can be used to form 1D metal oxide nanostructures. Biosensors in which such structures were applied exhibit enhanced sensitivity due to the high surface area, especially in hollow 1D nanostructures. The development of optical biosensors requires one to form nanostructures that can tune and enhance optical signal for the detection of target biological substances. In this case, 2D nanostructures or thin films that can be formed by the ALD method are frequently applied. Novel structures, a high surface-to-volume ratio, and the porosity of 3D nanostructures formed by the wet chemical method allowed to achieve an extremely low LOD and opened a new way of forming 3D nanostructures in electrochemistry-based chemical sensors and biosensors. Flower-like MoO_3_/In_2_O_3_ microstructures formed by the hydrothermal method and composed of numerous nanosheets are promising for the development of gas sensors [123].

However, there is still room for improvement and challenges related to different synthesis methods. Improvements and more research are required to form nanostructures without side effects and with the desired properties for the development of sensitive, reproducible, easy to use, and fast biosensors with minimal pollution of the living nature. The achievement of high stability and nontoxicity in various nanostructures formed from metal oxides, such as ZnO, TiO_2_ or SiO_2_, which are applied in vivo biosensing and are exposed by different liquid ambient, is still an issue. Therefore, we expect significant experimental and theoretical research activity regarding the synthesis methods for metal oxide nanostructures of different properties and application for sensing. 

## Figures and Tables

**Table 1:** Nanostructure formation methods, detected analyte and reported biosensing properties.

Nanostructure	Method of Synthesis	Application	Reported Sensing Properties	Ref.
ZnO nanostructures	Chemical bath deposition	Electrochemical immunosensor for ZIKV-NS1 antigen	LOD: 1.00 pg/mL LDR: 0.1–100 ng/mL	[100]
MoS_2_/Cu_2_O	Chemical vapour deposition, electrodeposition	GSH, GSSG biosensor for indirect cancer cell detection	LDR: 0–50,000 cells	[101]
Au(Fe_3_O_4_) MNPs	Co-precipitation, wet chemical	Colorimetric hGH sensor	LOD: 0.082 nM LDR: 0.1–5.0 nM	[102]
Au(Fe_3_O_4_) MNPs	Co-precipitation, wet chemical	SPR CD5 immunosensor	LOD: 8.31 fM	[103]
ZnO NRs-rGO	Electrodeposition	Electrochemical IgG detection	LOD: 1.25 ng/mL LDR: 10–1000 ng/mL	[67]
ZnO NWLs	Electrodeposition	Electrochemical enzymatic H_2_O_2_ sensor for cancer cell detection	LOD: 0.8 μM LDR: 1–1000 μM	[104]
Au/(TiO_2_/SiO_2_) Photonic crystal	Ion beam sputtering	TIRE signal enhancement	6.4 times more sensitive than SPR	[105]
Au/(TiO_2_/SiO_2_)/Au	Ion beam sputtering	SE/QCM-D signal enhancement	23 and 49 times increased SE signal	[106]
Ag/ZnO-NRs/Au	Thermal evaporation; hydrothermal; sputtering	SERS sensing platform	LOD (λ-DNA): 10 ng/μL LOD (Rh6G): 10^−16^ M	[107]
Au/PAA	Two-step anodization; thermal evaporation	Interferometric aptamer based bacterial cell sensor	LOD: 20 CFU/mL LDR: 10^3^–10^9^ CFU/mL	[108]

Abbreviations: ZIKV-NS1—Zika virus NS1 antigen, LOD—limit of detection, LDR—linear dynamic range, GSH—glutathione, GSSG—glutathione disulphide, MNP—magnetic nanoparticle, hGH—human growth hormone, SPR—surface plasmon resonance, CD5—lymphocyte antigen T1, NRs—nanorods, rGO—reduced graphene, IgG—Immunoglobulin G, NWLs—nanowalls, TIRE—total internal reflection ellipsometry, SE—spectroscopic ellipsometry, QCM-D—quartz crystal microbalance with dissipation, SERS—surface enhanced Raman spectroscopy, Rh6G—Rhodamine—6 G, PAA—porous anodic alumina, CFU—colony forming unit.

**Table 2:** Nanostructure formation methods, detected analyte, and reported chemical sensing properties.

Nanostructure	Method of Synthesis	Application	Reported Sensing Properties	Ref.
Ni(OH)_2_ HRs	Chemical bath deposition	Electrochemical glucose sensor	LOD: 0.6 μM LDR: 0.002–3.8 mM	[114]
ZnO NSs	Chemical bath deposition	Electrochemical aqueous formaldehyde sensor	LOD: 210 nM	[115]
ZnO NRs	Hydrothermal	Fluorescence thiabendazole sensor	LOD: 304 nM LDR: 10–80 μM	[116]
SiO_2_/GSCDs	Hydrothermal, reverse microemulsion	Fluorescence phenobarbital sensor	LOD: 0.1 nM LDR: 0.4–34.5 nM	[117]
Ag-TiO_2_ NPs/MWCNTs	Hydrothermal, powder blending	Electrochemical cetirizine sensor	LOD: 8.76 nM LDR: 0.3–3 μM	[118]
TiO_2_@SiO_2_ NSs	Sol-gel	Electrochemical ascorbic acid sensor	LOD: 383.3 μM LDR: 50–2500 μM	[119]
Lucigenin doped SiO_2_ NPs	Reverse microemulsion	Fibre optic Cl^−^ sensor	LDR: 0.02–0.06 M	[120]
ZnO/NiO/Al_2_O_3_ NPs	Wet chemical	Electrochemical L-glutamic acid sensor	LOD: 95.35 pM LDR: 0.1 nM–0.01 mM	[121]
Ni-ZnO NPs	Wet chemical	Electrochemical hydrazine sensor	LOD: 1.7 pM LDR: 0.2 nM–0.02 M	[122]

Abbreviations: HRs—hollow rods, LOD—limit of detection, LDR—linear dynamic range, NS—nanosheets, GSCDs—green source carbon dots, NPs—nanoparticles, MWCNTs—multi-walled carbon nanotubes.

## Data Availability

Not applicable.
